# Supplementary material for: Qualitative Evaluation of a Quality Improvement Collaborative Implementation to Improve Acute Ischemic Stroke Treatment in Nova Scotia, Canada
Source: Healthcare (Basel). 2024 Sep 10;12(18):1801. doi: 10.3390/healthcare12181801 (PMC11431084; doi:10.3390/healthcare12181801)
Supplement: Supplementary file 1 [file healthcare-12-01801-s001.zip › Supplementary File S1.pdf]

Supplementary File S1: COREQ Guidelines

Item	Question	Answer	Reference
**1. Interviewer/facilitator**	Which authors conducted the interviews or focus groups?	One interview was conducted by Shadi Aljendi and Kelly J. Mrklas. All other interviews were conducted by Shadi Aljendi.	Page 6
**2. Credentials**	What were the researchers' credentials?	Shadi Aljendi and Noreen Kamal have Ph.D. degrees, and Kelly Mrklas was in the final stages of finishing her Ph.D.	Page 6
**3. Occupation**	What was their occupation at the time of the study?	Noreen Kamal was an Associate Professor. Shadi Aljendi was a research engineer, and Kelly Mrklas was a final-year Ph.D. student.	Page 6
**4. Gender**	What was their gender?	The research team included both women (NK and KM) and men (SA) members.	For SA- Page 6: Accordingly, those participants who attended these meetings may have known his role in the project.<br>For KM- Page 6: while KM was in the final stages of completing her Ph.D.<br>For NK- Page 8: NK implemented the mQIC intervention but she did not participate in data collection or analysis for this study

**5. Experience and training**	What was their experience or training in qualitative research?	The team had extensive experience in qualitative research, particularly Kelly Mrklas, who had over 15 years of experience in conducting and assisting qualitative studies.	Page 25
**6. Relationship established**	Was a relationship established prior to study commencement?	The research team had an established relationship between each other prior to study commencement.	In Page 1, both NK and SA work in the same department. In Page 26, KM was a co-author of reference 16.
**7. Participant knowledge of the interviewer**	What did the participants know about the researcher?	Noreen Kamal had built rapport with the participants. Shadi Aljendi attended workshops and webinars, but no relationship was established. Kelly Mrklas had no relationship with the participants.	For NK- Page 25: NK conceived of the ACTEAST project, obtained funding for it, managed all the activities<br>For SA and KM - Page 6
**8. Interviewer characteristics**	What characteristics were reported about the interviewer/facilitator?	The interviewers were described as empathetic, non-judgmental, and skilled at facilitating open discussions.	Not mentioned in the manuscript
**9. Methodological orientation and theory**	What methodological orientation was stated to underpin the study?	The study was based on the CFIR framework analysis.	Page 6
**10. Sampling**	How were participants selected?	Interviewees volunteered after a call was distributed to all project participants. They were recruited via email through their participation in the quality improvement intervention.	Pages 5 and 6

**11. Method of approach**	How were participants approached?	Participants were approached via email to participate in Zoom video call interviews, with one conducted by phone at the interviewee's request.	Pages 5 and 6
**12. Sample size**	How many participants were in the study?	The study included 14 participants from 10 different entities.	Page 8
**13. Non-participation**	How many people refused to participate or dropped out?	No one refused, but some did not respond to the invite emails. No participants dropped out after consenting.	Not directly mentioned in the manuscript
**14. Setting**	Where was the data collected?	Data were collected at the participants' workplaces and through virtual interviews.	Page 6
**15. Presence of non-participants**	Was anyone else present besides the participants and researchers?	No, only the participants and researchers were present.	Page 6
**16. Description of sample**	What are the important characteristics of the sample?	The sample included healthcare professionals with varied roles and experience in stroke care.	Page 8
**17. Interview guide**	Were questions, prompts, guides provided by the authors?	Yes, a semi-structured interview guide based on CFIR was provided.	Page 5
**18. Repeat interviews**	Were repeat interviews carried out?	No, repeat interviews were not conducted.	Page 6
**19. Audio/visual recording**	Did the research use audio or visual recording?	Yes, all interviews were audio-recorded for accuracy.	Page 6
**20. Field notes**	Were field notes made during and/or after the interview or focus group?	Yes, field notes were taken during and after each interview.	Page 6
**21. Duration**	What was the duration of the interviews or focus groups?	Each interview lasted between 22 and 52 minutes.	Page 8

**22. Data saturation**	Was data saturation discussed?	Yes, data saturation was discussed.	Page 24
**23. Transcripts returned**	Were transcripts returned to participants for comment and/or correction?	No, transcripts were not returned due to participants' heavy workloads during the COVID-19 pandemic.	Page 6
**24. Number of data coders**	How many data coders coded the data?	Initially, all three researchers coded the data until consensus was reached, then Shadi Aljendi continued with regular verification from Noreen Kamal.	Page 6
**25. Description of the coding tree**	Did authors provide a description of the coding tree?	Yes, a detailed coding tree was developed based on CFIR constructs.	Pages 10 and 11
**26. Derivation of themes**	Were themes identified in advance or derived from the data?	Themes were derived deductively using the CFIR framework.	Page 6
**27. Software**	What software, if applicable, was used to manage the data?	NVivo software was used to manage and analyze the qualitative data.	Page 6
**28. Participant checking**	Did participants provide feedback on the findings?	No, transcripts did not provide feedback due to participants' heavy workloads during the COVID-19 pandemic.	Page 6
**29. Quotations presented**	Were participant quotations presented to illustrate the themes/findings?	Yes, participant quotations were used to illustrate the themes.	Pages 12 - 20
**30. Data and findings consistent**	Was there consistency between the data presented and the findings?	Yes, there was consistency between the data and the reported findings.	Page 21 -23

**31. Clarity of major themes**	Were major themes clearly presented in the findings?	Yes, major themes were clearly presented and supported by data.	Pages 12 - 20
**32. Clarity of minor themes**	Is there a description of diverse cases or discussion of minor themes?	Minor themes were also presented in the results table.	Page 10
