# Supplementary material for: Qualitative Evaluation of a Quality Improvement Collaborative Implementation to Improve Acute Ischemic Stroke Treatment in Nova Scotia, Canada
Source: Healthcare (Basel). 2024 Sep 10;12(18):1801. doi: 10.3390/healthcare12181801 (PMC11431084; doi:10.3390/healthcare12181801)
Supplement: Supplementary file 1 [file healthcare-12-01801-s001.zip › Supplementary File S2.pdf]

Supplementary File S2: Interview Guide

Interview Questions

Preamble: This interview is covered by the consent form that you signed at the beginning of the ACTEAST initiative. This study is approved by NS Health's Research Ethics Board, and this approval includes the consent form. The consent form ensures that your participation is voluntary, and you can stop this interview at any time.

The interview will be recorded and if you'd like to say something that you know you don't want recorded, just let us know and we can flip the recording off.

You will not be identified in any publications or other research outputs. We can provide a copy of your consent form if you wish to review it.

General Questions

1. What gender do you identify with?

2. What hospital or site do you work in?

3. What is your position and/or role at this hospital?

Feedback on the ACTEAST Initiative

Please rate your answers to these next questions on a scale from 1-5 where 1 is strongly disagree and 5 is strongly agree. If you would like to add information to clarify your answer, please feel free to do so after your response:

4. **Putting together an improvement team** for the ACTEAST Initiative had a significant impact on improving acute stroke treatment at your hospital. State your level of agreement from 1 to 5 (1 is strongly disagree and 5 is strongly agree).

1	2	3	4	5	DK
Strongly disagree	Disagree	Neither agree nor disagree	Agree	Strongly agree	Don't Know

   a. Why? Why not? Explain.

5. **Showing the evidence** for acute stroke treatment in the Learning Session presentations had an impact on improving acute stroke treatment at your site. State your level of agreement from 1 to 5 (1 is strongly disagree and 5 is strongly agree).

1	2	3	4	5	DK
Strongly disagree	Disagree	Neither agree nor disagree	Agree	Strongly agree	Don't Know

   a. Why? Why not? Explain

6. **Showing changes happening at other sites** during the Learning Session presentations had an impact on improving acute stroke treatment at your site. State your level of agreement from 1 to 5 (1 is strongly disagree and 5 is strongly agree).

1	2	3	4	5	DK
Strongly disagree	Disagree	Neither agree nor disagree	Agree	Strongly agree	Don't Know

   a. Why?

7. The **Action planning** you did with your team at the Learning Sessions had an impact on improving acute stroke treatment at your site. State your level of agreement from 1 to 5 (1 is strongly disagree and 5 is strongly agree).

1	2	3	4	5	DK
**Strongly disagree**	**Disagree**	**Neither agree nor disagree**	**Agree**	**Strongly agree**	**Don't Know**

a. Explain.

b. Were you involved in the action planning? (this will apply if the response is "Don't Know")

c. Was the action plan used?

8. **The virtual site visit** had an impact on improving acute stroke treatment at your site. State your level of agreement from 1 to 5 (1 is strongly disagree and 5 is strongly agree).

1	2	3	4	5	DK
**Strongly disagree**	**Disagree**	**Neither agree nor disagree**	**Agree**	**Strongly agree**	**Don't Know**

a. Explain.

9. **The webinars conducted during action periods** had an impact on improving acute stroke processes at your site. State your level of agreement from 1 to5 (1 is strongly disagree and 5 is strongly agree).

1	2	3	4	5	DK
**Strongly disagree**	**Disagree**	**Neither agree nor disagree**	**Agree**	**Strongly agree**	**Don't Know**

a. Explain.

10. The **data feedback** you received from ACTEAST had an impact on acute stroke treatment at your site. State your level of agreement from 1 to 5 (1 is strongly disagree and 5 is strongly agree).

1	2	3	4	5	DK
**Strongly disagree**	**Disagree**	**Neither agree nor disagree**	**Agree**	**Strongly agree**	**Don't Know**

a. Explain.

*For the following three questions, please reflect on your **ACTEAST Initiative experience**:*

11. What was the **most beneficial** part of the Improvement Collaborative?
12. What was the **least beneficial** part of the Improvement Collaborative?
13. Can you tell me if the effectiveness of the ACTEAST Initiative was influenced by the virtual/online format of communication? Please explain.

14. On a scale of 1 to 5, where 1 is very easy and 5 is very difficult, based on your experience, how easy or difficult was it to **participate** in the ACTEAST Initiative?

1	2	3	4	5	DK
**Very easy**	**Easy**	**Neither easy nor difficult**	**Difficult**	**Very Difficult**	**Don't Know**

a. Please explain.

15. On a scale of 1 to 5, where 1 is very easy and 5 is very difficult, based on your experience, how easy or difficult was it to **implement** the process changes?

1	2	3	4	5	DK
**Very easy**	**Easy**	**Neither easy nor difficult**	**Difficult**	**Very Difficult**	**Don't Know**

a. Please explain.

Feedback on Improvements at Your Site

16. What is your role in the acute stroke treatment process? And what was your specific role in the ACTEAST Project?

17. What (if any) skills related to the acute stroke treatment process did the ACTEAST Initiative help you to build/improve?

18. Looking back, can you tell me if your **understanding of the evidence** for acute stroke treatment changed through the improvement collaborative? If it changed, can you tell me how?

19. If you had a 1 min elevator ride to tell a colleague about what the evidence and guidelines say about delivering tPA and EVT for acute ischemic stroke, what would you say?

20. Was there a strong need or desire to improve the acute stroke treatment process at your site before ACTEAST? (prompt: the need or desire to improve could come from the hospital, health authority or clinicians.) Please explain.

21. What kinds of changes did you and your team make to the acute stroke treatment process at your hospital?

22. Were there any changes that your hospital was **not able to make**?

    a. Are there any changes that your hospital still plans to make?

23. Hospital culture can sometimes influence how a new intervention is implemented. Can you tell me about the culture at your site?"

    a. How do you think this culture influenced the ACTEAST Initiative?

    b. From your perspective, how welcoming or conservative was the team/site staff/leadership to adopting a new intervention?

24. What influence did other hospitals' performance have on your improvement efforts?

25. These **next questions are about communication and information.** Can you tell me how you typically find out about things related to this project?

26. Can you tell me how you typically find out about things in general among your team with regard to stroke treatment?

27. In your opinion, how well did your improvement team work together?

28. How did you deal with competing priorities at your hospital?

ACTEAST
Atlantic Canada Together
Enhancing Acute Stroke Treatment

29. In your opinion, how important is getting the patient/patient's family consent prior to treatment?

30. After participation in the ACTEAST Initiative, are you confident in making changes to the stroke treatment process? (YES/NO/Don't Know)

31. Were any steps taken at your site to ensure team members made practice and process changes?

32. Did you feel supported to try new things?

33. How were you supported during the ACTEAST Initiative? Were there any special recognitions or rewards planned that are related to implementing the intervention?

34. Were you able to get key influential individuals on board with the improvement initiative?

35. Since the improvement collaborative ended:

    a. What parts of the initiative do you think will stay in place?

    b. What do you think will be hard to keep up?

    c. Are there parts that you still consider 'extra to your daily work', or parts that are still hard to get integrated? If so, which ones?
