# Supplementary material for: Qualitative Evaluation of a Quality Improvement Collaborative Implementation to Improve Acute Ischemic Stroke Treatment in Nova Scotia, Canada
Source: Healthcare (Basel). 2024 Sep 10;12(18):1801. doi: 10.3390/healthcare12181801 (PMC11431084; doi:10.3390/healthcare12181801)
Supplement: Supplementary file 1 [file healthcare-12-01801-s001.zip › Supplementary File S3.pdf]

Supplementary File S3: Extra Citations

3.1.1. (D1.2) Evidence Strength & Quality

Interviewee003 about certain categories of healthcare professionals not having been convinced of the evidence: "even though the emergency physicians are not part of the process, or there is a very small part of the process because they activate the stroke protocol and then they basically step back. I think we're still influenced by their attitudes towards [Tissue Plasminogen Activator] (tPA). And not all of them buy in to the fact that tPA is helpful, and, some of them feel it's harmful and so that may influence some of the activations"

Interviewee001 about the benefit of presenting the evidence: "it's always good to review and kind of put it into context. I would say that probably, for the nurses or admin people on the team, it might have been newer information for them too, and it could help them to understand the rationale for what we were doing".

Interviewee002 about the change in the understanding of the evidence: "those presentations in particular really expanded my understanding of the evidence around imaging and its impact in decision making".

3.1.2. (D1.3) Relative Advantage

Interviewee010 about one benefit of ACYEAST is that it initiated conversations: "I think this initiative provided us with the framework for launching at those initiatives and then opportunities of learning more from this research"

Interviewee012 about how the improvement team and the principal investigator discussed specific improvements that made the process more efficient and improved the DNT and stroke care "it kind of brought it to the forefront. So it's not just, it's another stroke, it's OK, let's see what we can do. Let's see what we can save. You know how much you know about disease we can [improve processes to avoid] long lasting disability".

Interviewee010 about how ACTEAST provided a framework for launching initiatives planned before the project started. For example, "I think this initiative provided us with the framework for launching at those initiatives".

Interviewee010 about how ACTEAST enabled communication between sites and fostered broader discussions around the benefits and risks of lytics versus EVT: "[ACTEAST] fostered broader discussions around the benefits and risks of lytics versus EVT and imaging".

Interviewee013 about the advantage of monitoring and analyzing data to determine where improvements must occur: "then to start to really begin to critically appraise the work that we're doing and figure out where we are not achieving our goals".

Four interviewees appreciated the design quality of the project, as all the necessary information was provided with check-ins from the Principal Investigator. For example, interviewee007 said "all the information was provided. Things were set up for us. [Principle Investigator] checked in on things".

3.2.1 (D2.1) Needs & Resources of Those Served by the Organization

Interviewee002 pointed to how ACTEAST brought the question of consent to the forefront of discussion "And this was another not an issue at our site, but kind of something that our physicians had some questions about".

Two interviewees stated that calling 911 or presenting to a hospital is an indirect form of consent. Interviewee010 says "From a paramedic perspective, we assume that they're consenting their care by calling 911".

Interviewee013 about getting consent: "We don't get consent to treat people who have been in a trauma. We don't get consent to treat people if they've had a heart attack".

This aspect significantly influences the direct care of stroke patients. The assertion that consent profoundly impacts direct care of stroke patients is substantiated by the qualitative analysis of interviews, such as the insight from Interviewee003 who states "I don't think informed consent in the traditional sense is practical in that situation, because it is very much an emergency situation. And the longer you delay it, the worse they're going to do".

3.2.2 (D2.2) Cosmopolitanism

Interviewee003 about how ACTEAST was an opportunity for older participants to get to meet and know newly joined ones: "there are a lot of players that have changed in that time and I may know the stroke coordinator in an area, but they don't necessarily know the physicians, and especially the emergency physicians. So, it was really good to see all that engagement around the province. I think that was probably a big benefit from [ACTEAST]".

Three interviewees highlighted the "invaluable" ACTEAST networking opportunities. Interviewee004 said "Those networking opportunities are invaluable".

This includes local specialists that would be collaborators on some future cases. Interviewee013 mentioned that "The idea that we're all in this together and that we need to work collaboratively".

3.2.3 (D2.3) Peer Pressure

Four interviewees stated that seeing changes in other sites created positive competition. This allowed sites to learn from the experiences of other sites, both positive and negative. Interviewee008 said "hearing how one of the sites in our province had their door to tPA time down to 10 minutes with just incredible because we were very impressed with that and we're doing what we could to try and improve our time to get closer to theirs".

Seeing other sites' performance data and changes was positive for nine interviewees. For example, Interviewee005 said "I found it really gave our site new ideas to try to improve times at our own site".

According to interviewee004, knowing what is going on at the different sites is essential for the tertiary site in terms of realizing that the referring sites are doing what is required to prepare patients before being transferred. They mentioned that "especially when we're referring to Halifax as our tertiary care site, you can see what they're doing and what we can do to get things ready for patients going there".

Interviewee006 highlighted that the overload resulting from the COVID-19 pandemic has diminished the positive effects of peer pressure by saying "just because of time and you know people not being able to

actually enact anymore change because they had no time to do it because they were in this COVID meeting or in that COVID meeting”.

Another interviewee pointed out that sites in their particular region do not like to be compared to other places, and it was tricky to consider peer pressure as positive motivation. However, the sites that had a peer pressure impact were in the same geographical zone with similar resources and sizes. Interviewee002 said “I think in [our province] we're a little bit unique, whereas when we see those numbers we kind of get our defenses up and we think we don't love to be compared to other places”. They continue to say that a certain site “is in our zone and resource wise they are relatively similar to us, so I think it's easier for folks to make the relationship between numbers there and numbers here”.

3.3.1 (D3.2) Networks & Communications

Networks and communication challenges were mentioned by three interviewees. Interviewee001 mentioned at the beginning of the project “identifying the team was challenging”. Another challenge was the disjointed teamwork at times according to Interviewee003 who said “COVID really interfered with the ability to meet and I think, had we been able to meet, maybe every two weeks in person as a group, it could have gone a lot smoother”. Interviewee005 explained how COVID-19 restrictions, and significant team change within the first couple of months negatively affected the participation in the mQIC “they were over committed, and so there was a change because some people backed out”.

On the other hand, a principal part of ACTEAST was the development of multidisciplinary improvement teams and the facilitation of collaboration within the team. These were well appreciated by four interviewees. Interviewee001 said “I feel like the most beneficial part [of ACTEAT] was the fact that we had to develop the team”.

A concrete example of this kind of difficulty was given by Interviewee012 who said “in meetings with our imaging department, things like trying to convince the radiologists to call the ED doc right after the image is done. Something as simple as that. Yes, we can't do that”. Interviewee013 described the culture at their site to be “the culture of a busy, highly stressed emergency department has challenged the ACTEAST process to become more efficient”.

ACTEAST helped culture to change over time at one site and became very open. Interviewee001 said that the culture “has probably changed a little bit over time [to become] open and willing to try new things”. Intervewee009 explains how most of the younger and nursing staff were open to change while doctors were not by saying “So I think that the nursing staff on the most part and the extra staff were open to a little bit of change. Doctors not so much. And then the also, the younger people younger staff were open to changes, [so] culture is better”.

Adopting changes was welcomed by the teams and leadership at five sites. For example, Interviewee007 pointed to how the change of the leadership led to a more open culture “We had a bit of a change of leadership in the middle, so nearer to the end, everyone was very welcoming”. However, even with everybody onboard, changes still needed a lot of collaboration within sites and accordingly dealing with each sites' own cultures, according to Interviewee010 who said “a lot of collaboration was needed with sites and working with other kind of settings and their cultures and how care is provided”. Moreover, Interviewee007 said “if a process change impacts a lot of departments, there can be a lot of risk in that,

and so people are adverse to just jumping into something that does have more global impact. So we do have processes clearly set up to implement change appropriately”.

However, to EHS, despite some challenges, change is welcome. Interviewee007 stated, "prehospital care is an ever-changing environment, so we are always willing to change, but don't love change". In addition, Interviewee010 says “within Paramedicine in Nova Scotia we have a focus on evidence based medicine and we have a history of collaborations with emergency medicine”.

3.3.3.1 (D3.4.3) Relative Priority

At other sites, individual role tasks of some participants were prioritized over some ACTEAST activities. Interviewee003 pointed that “part of the issue is that the people who are on these teams are typically the people who are on lots of teams […] so they have lots of things on their table”.

COVID-19 and the different pandemic waves were getting the highest priority at five sites. Interviewee002 said “engagement was a little bit tough in part because people had their priorities [during the pandemic lockdown]”. One interviewee thought that the pandemic priorities were even used as an excuse for not achieving improvement in reducing DNT. The extra load resulting from COVID-19 led interviewee006 to a feeling of failure in dealing with competing priorities as they say “Towards the end it was a major issue and I feel like I was drowning”.

According to four interviewees, dedicating staff to managing the ACTEAST activities was the way to keep it prioritized. For example, Interviewee012 says “having [someone] dedicated to it […] was amazing”.

At three sites, stroke always had the highest priority. For example, Interviewee004 says “I didn't really find any competing priorities”.

3.3.3.2 (D3.4.5) Goals and Feedback

One interviewee explained how it boosted the team's morale, generated ideas, and engaged new participants and noticed that specific site struggles and solutions were discussed during the visit by saying “it kind of had a boost on the morale of the team and I think it gave them some ideas of things because it's one thing in a large group session when people are talking about generic ideas that can be done, but it is helpful when somebody [principal investigator] either face to face or virtually is visiting directly with you learning about the struggles you're having, the problems you're having and coming up with specific solutions for those”. However, for interviewee001, the virtual visit was the most challenging part of the project to engage people with as it was hard to understand its benefit. Interviewee001 said “This was probably the piece of the project that I struggled to get the most people engaged with […], maybe it was harder for us all to understand the benefit of this piece”. Accordingly, interviewee001 reported that the virtual visits were not impactful.

3.3.4 (D3.5) Readiness for Implementation

Leadership engagement was critical for implementation, with some interviewees highlighting the importance of support from higher positions such as Interviewee001 who said “I mean obviously if they [the managers] didn't support it would have been hard to move ahead with it”. Other interviewees emphasized a collective leadership approach as was the case with Interviewee004 who said “I mean, they just want us to do what you need to do. Let us know if you need some help so there's no issues anywhere along the way”.

Time was a scarce resource, often due to competing commitments like COVID-19. Interviewee002 said "there were times when I felt like I was asking a lot of other people, so I would just do it myself and then it was a lot for myself", and Interviewee005 said, "I think people are busy and changes to the system take time. You need time to educate and then you need to evaluate the things and then change again if needed". Solutions included task prioritization and efficient planning.

3.3.4.1 (D3.5.1) Leadership Engagement

The interviewees felt that leadership engagement had a significant impact on their results. For interviewee013, the mQIC did not result in a significant impact because the team could not engage people in higher positions with authority to implement the planned changes. Interviewee003 said "one of the other reasons it didn't have a big impact is that we were not able to engage other people and mostly people in higher positions who have the authority to implement the changes that we want to change". Conversely, one interviewee mentioned that prior roles and good relationships with their leadership resulted in getting the buy-in from leadership and everyone down as Interviewee006 says "because of some prior roles that I've had, I have a good relationship with the leadership at the hospital anyway, so we had buy-in from the site lead at the hospital and really from everyone on down". On the other hand, Interviewee013 felt that leadership is provided by all employees, "We are the leadership. I must say we don't spend a lot of time talking to people who work at levels above us just because they don't have the power to make to effect change". In addition, Interviewee014 explained that there is no need for processes that go through several levels of authority in a small hospital by saying "We don't need to multi-level processes, we decide what we're going to do between us and the emergency department and the internists and we are able to implement those without bureaucracy".

3.3.4.2 (D3.5.2) Available Resources

One interviewee expressed that some participants, physicians in particular, already had busy schedules as mentioned by Interviewee003 "So [the mQIC] was difficult to fit into an already very busy schedule". Time was limited because some participants volunteered for many projects or the COVID-19 extra meetings.

To work around the busy schedule, prioritizing tasks and a task-oriented operation with specified deadlines were adopted as per Interviewee004. The same interviewee recognized the excellent planning and organization of the mQIC allowed for a smoother time commitment. Other mentions of limited resources by three interviewees included limitations of financial resources, particularly for a stroke coordinator position and other required material. For example, Interviewee003 said "it was a lot of things that we need significant financial or other resources to implement".

3.3.4.3 (D3.5.3) Access to Knowledge and Information

According to Interviewee009, the information and knowledge acquired through the project resulted in tuning protocols to the sites' particular needs. The interviewee says "fine tuning a protocol to your site specifically that was set for me".

The webinars conducted during the mQIC were a source of knowledge and information. According to six interviewees, the topics covered in the webinars were excellent, even with varied attendance because experts were available in webinars for comments and questions, even when they were not presenting. For example, interviewee001 said "I thought the topics that were covering were important. We didn't always

have great attendance that are from our sites. It kind of varied, but what was good about them was that though, it's like learning what's happening to other sites, but also having the experts available for comments and questions. So even if the you know experts weren't presenting, you had neurology or interventional neuroradiology on the call, making comments and answering questions”.

Two interviewees explained that the topics and conflicting meetings presented challenges to attending the webinars. For example, Interviewee006 said “I missed a number of these [webinars] because of having to be at conflicting meetings”. Interviewee002 suggested having external experts presenting, as opposed to local, as local initiatives can sometimes be limited in their progress. The interviewee said “sometimes local initiatives can't be progressed further. Unless you've got expert coming in saying this is where we need to go”.

3.5.1 (D5.1) Planning

Interviewees thought the action planning was impactful even though not perfect because of COVID-19, according to interviewee005 who said “not that it was perfect either because again, same caveats as previously with COVID really throwing a wrench in some things”. For Interviewee009, it helped focus efforts and provided a framework and a list of actions. The interviewee said “It did help focus our efforts and provide us with that framework for improving our care over that six month period. Gave us a list of things we wanted to work on”. Interviewee007 explained how having several departments at the table with the right stakeholders involved allowed for better process streamlining. The interviewee said “different departments at the table who were all invested in this, it let us develop some processes a little more streamlined and with the right people involved and the right stakeholders. And because of that we were able to move some changes along faster than we would have if we didn't have the collaborative”. At Interviewee013’s site, different communication and coordination issues resulted in inefficient action plan, as the interviewee explained “They came up with things that needed to be fixed. Then for some things, they determined actions that should be taken. Didn't really assign them to people and they didn't really set a date when they should be done”.

3. 5.2.1 (D5.2.5) Key Stakeholders (Staff)

According to four interviewees, appropriate participants were able to influence their departments. For example, Interviewee001 said “we had appropriate people that are sitting at […] our tables and so if you got the buy in from everyone at the table, really they went back to their own department or specific team in the hospital”. Interviewee001 mentioned that the mQIC had created an environment where everyone felt efficient. The interviewee said “I feel like everyone that was involved was influential on their own right because they really pushed things in their own departments”. For two interviewees, engaging emergency staff and EHS physicians was a key to success. For example, Interviewee012 said “the biggest thing is the engagement from who needs to be engaged from EHS to DI to the physicians to the staff, you know, so it's just reaching out and you know making sure everybody has what they need and has the information they need to get them there”.

3.5.3 (D5.4) Reflecting & Evaluating

Reflection and evaluation of the mQIC showed mixed feelings about the mQIC. For Interviewee005, everything related to the ACTEAST mQIC was stressful due to added daily work. The interviewee said

about the mQIC "It was almost like adding another, you know 25% onto an already 100% job that's you know. So there were times that it left me stressed".

Interviewee013 was a stroke neurologist and all the ACTEAST mQIC activities and tasks were part of their daily job. Still, for that same interviewee, sustaining the changes and operationalizing the work was challenging. Interviewee013 reported that further followup after the mQIC ended and continuous data feedback would be excellent by saying "be nice to follow up with the [Principle Investigator] and kind of hear, next steps".
